# Protective effects of *Fraxinus xanthoxyloides* bark in alloxan-induced diabetic rats: A phytochemical and pharmacological approach

**DOI:** 10.1371/journal.pone.0346328

**Published:** 2026-06-04

**Authors:** Mashal Shahzadi, Tahira Younis, Ayman M. Al-Qaaneh, Ali Raza Ishaq, Farah Deeba, Musrat Shaheen, Mostafa A. Abdel-Maksoud, Saeedah Almutairi, Abdulaziz Alamri, Heba A. S. El-Nashar

**Affiliations:** 1 Department of Zoology, Faculty of Life Sciences, Government College University Faisalabad, Faisalabad, Pakistan; 2 Department of Biochemistry and Biotechnology, Faculty of Life Sciences, The Women University Multan, Multan, Pakistan; 3 Faculty of Allied Medical Sciences, Al-Balqa Applied University (BAU), Al-Salt, Jordan; 4 State Key Laboratory of Biocatalysis and Enzyme Engineering, Environmental Microbial Technology Center of Hubei Province, College of Life Science, Hubei University, Wuhan, China; 5 Institute of Microbiology, Faculty of Life Sciences, Government College University Faisalabad, Faisalabad, Pakistan; 6 Research Chair of Biomedical Applications of Nanomaterials, Biochemistry Department, College of Science, King Saud University, Riyadh, Saudi Arabia; 7 Botany and Microbiology Department, College of Science, King Saud University, Riyadh, Saudi Arabia; 8 Biochemistry Department, College of Science, King Saud University, Riyadh, Saudi Arabia; 9 Department of Pharmacognosy, Faculty of Pharmacy, Ain Shams University, Cairo, Egypt; 10 Department of Pharmacognosy, Faculty of Pharmacy, Modern University for Technology & Information, Cairo, Egypt; Institute of Medical Sciences, Banaras Hindu University, INDIA

## Abstract

*Fraxinus xanthoxyloides* Wall. ex DC (Family-Oleaceae) is a tiny tree found in arid highlands, often referred to as “Afghan ash”. This study aimed to explore *F. xanthoxyloides* bark extract as a potential anti-diabetic agent by conducting GC-MS analysis along with *in vitro* and *in vivo* studies. *F. xanthoxyloides* bark methanol extract (FXBM) was fractionated with n-hexane (FXBH), followed by chloroform (FXBC), ethyl acetate (FXBE), and residual aqueous fraction (FXBA). GC-MS and HPLC-MS analysis of the total extract were performed. The inhibitory activities against α-amylase, α-glucosidase, and DPP4 were assessed for the extract and its fractions. The *in vivo* study included five groups: control group, diabetic control, groups pretreated with alloxan (150 mg/kg) + glibenclamide (5 mg/kg), group pretreated with alloxan (150 mg/kg) + FXBH (200 mg/kg), and group pretreated with alloxan (150 mg/kg) + FXBH (400 mg/kg). Further, a histopathological investigation was conducted. GC-MS analysis of FXBM revealed the presence of 17 compounds predominated by esters (17.9%), polyols (16.35%), and *O*-glycosides (12.24%). Among all extract/fractions, FXBH showed maximum α-amylase, α-glucosidase, and DPP4 inhibition when compared to acarbose and berberine, respectively. It also achieved the highest glucose uptake among all extract/fractions when compared with metformin. HPLC-DAD analysis showed the presence of gallic acid (3.29 µg/mg), catechin (4.23 µg/mg), caffeic acid (6.05 µg/mg), ferulic acid (2.99 µg/mg), and quercetin (6.4 µg/mg). FXBH-treated rats showed a significant increase in body weight and reduced blood glucose levels (*p* < 0.05) from days 1–30. The biochemical parameters like triglyceride, low-density lipoprotein, cholesterol, lipase, amylase, C-reactive protein, alanine aminotransferase, aspartate aminotransferase, creatinine, HbA1c, and urea were decreased, while high-density lipoprotein was elevated, compared to diabetic control. Histopathological studies demonstrated restoration of pancreatic β-cells. In conclusion, *F. xanthoxyloides* bark extract exhibited potential antidiabetic effects; meanwhile, further studies are recommended to characterize the pharmacodynamics, pharmacokinetics, and synergistic effects with standard drugs.

## 1. Introduction

Diabetes mellitus (DM) is a serious global health concern affecting people regardless of country, age, and sex [1,2]. A recent investigation displayed that 463 million people of the world population (9.3%) have been affected by diabetes mellitus [3,4]. Moreover, it has been estimated that this number of affected people is going to escalate to about 578 (10.2%) by 2030 and 700 million (10.8%) by 2045 [5,6]. Pakistan (30.8%), French Polynesia (25.2%), Kuwait (24.9%), Nauru (23.4%), New Caledonia (23.4%), Northern Mariana Islands (23.4%), Marshall Islands (23.0%), Mauritius (22.6%), Kiribati (22.1%), and Egypt (20.9%) are the top ten countries with the highest prevalence of diabetes, according to the World Population Review Survey [1].

DM is a persistent, complicated metabolic syndrome that is characterized by increased glucose levels in the blood resulting from abnormal insulin production and function [7–9]. DM is of four types: type 1 diabetes mellitus (T1DM), type 2 diabetes mellitus (T2DM), gestational diabetes mellitus (GDM), and secondary diabetes mellitus [7,10]. The latest research has proved that T1DM is mainly caused by the destruction of insulin-producing β-cells of islets of Langerhans due to autoimmune mechanisms. The activation of cellular stress in pancreatic β-cells, which have a weak defense system, ultimately affects the synthesis of its vital hormone [11,12]. In contrast, T2DM affects 90–95% of the population of the world in which a person has insulin resistance for a long time due to the absence of β-cell insulin secretion [13,14]. GDM is an imbalanced glucose tolerance usually in the first pregnancy period that showed prevalence from region to region, with Southeast Asia having a prevalence of 25%, the Middle East and North Africa 17.5%, Europe 12.6%, and North America and the Caribbean 10.4% [15]. Given the serious burden of diabetes, it is essential to identify more effective therapeutic agents and treatment strategies.

Gut-derived peptides known as incretin hormones include GLP-1 and GIP, which are released by intestinal L and K cells, correspondingly. These hormones are released after feeding and have several physiological effects. These include increasing production of insulin against elevated glucose, promoting β-cell growth, and inhibiting glucagon release [16]. Despite the expression of the proglucagon gene in pancreatic and enteroendocrine L-cells [17], GLP-1 is synthesized in the intestine by posttranslational modification of proglucagon by prohormone convertase [18]. The realization that GLP-1 and GIP are quickly broken down by DPP-4 has spurred the development of specific protease inhibitors. These inhibitors are intended to avoid the quick decrease of GLP-1 levels in the blood stream following a meal [19]. Antidiabetic drugs currently used to treat type 2 diabetes aid in the regulation of blood glucose levels through several mechanisms: they can enhance insulin secretion, improve insulin sensitivity, reduce glucose absorption in the intestine, and/or increase glucose uptake in the peripheral system. Several classes of hypoglycemic agents currently available include insulin secretagogues, insulin sensitizers, and α-glucosidase inhibitors such as miglitol and acarbose. [20]. New peptide analogues that imitate the effects of incretin hormones are known as incretin mimetics. Examples include exenatide and liraglutide. Additionally, there are incretin enhancers, which are DPP-4 inhibitors, for instance, vildagliptin and sitagliptin, which have shown effectiveness in treating patients of type 2 diabetes [21].

Natural products are an everlasting source of phytochemicals that have been utilized by humans as natural medicines [22,23]. Likewise, Reza et al. (2020) proved that the plant extract of *Aeginetia indica* is a promising herbal medicine to treat alloxan-promoted diabetes as well as paracetamol-induced hepatotoxicity in mice [24]. Moreover, Xu et al. (2022) displayed that the fruit acetone extract of *Momordica charantia* is valuable for lowering blood glucose (13–50%) after 8–30 days of regular treatment. The compounds of *Momordica charantia* improve the insulin resistance by inhibiting IRS1 and JAK2 pathways [25]. Consumption of *Fraxinus excelsior* seed extract resulted in a considerable decrease in the glycemic increase that occurred after meals in diabetic individuals [26]. Obese mice were given *F. excelsior* seed extracts, which reduced their weight growth and hyperglycemia [27]. The literature reports that the genus *Fraxinus,* which is rich in phytochemicals like phenylethanoids, flavonoids, secoiridoids, lignins, and coumarins, has anticancer [28], renoprotective [29], antioxidant [30], anti-inflammatory [31], anti-analgesic [31], cardioprotective [32] and antidiabetic agents. *In vitro* studies have demonstrated that the chloroform fraction derived from the leaves of *Fraxinus xanthoxyloides* effectively inhibits the synthesis of NFκB induced by TNF-α and the synthesis of nitric oxide induced by LPS [31].

The traditional medicinal plant “*Fraxinus xanthoxyloides* (G.Don) Wall. ex A.DC. (family *Oleaceae*)" is widely distributed in Algeria, India, Pakistan, and Morocco [33]. Different parts of this plant are used by indigenous healers in Pakistan to treat patients with jaundice, pneumonia, and malaria [31]. Its stem bark is used by the locals to make a decoction that helps with bone fracture and labor pain [34]. The goal of this work was to examine the antidiabetic potential of the bark of *F. xanthoxyloides* against alloxan-induced diabetic rats. To the best of the author's knowledge, the antidiabetic properties of this plant have not yet been assessed. In the current study, we have evaluated the antidiabetic effect of *F. xanthoxyloides* bark in alloxan-induced diabetic rats both *in vivo* and *in vitro*.

## 2. Materials and methods

### 2.1. Plant material

*Fraxinus xanthoxyloides* bark was harvested in October 2023 from Quaid-i-Azam University (QAU) in Islamabad, Pakistan. To recognize the plant, we get assistance from Dr. M. Zafar from the Department of Plant Sciences, QAU Islamabad. A voucher specimen of the plant (45881) was deposited at the National Herbarium of QAU, Islamabad, Pakistan.

### 2.2. Extract preparation

After being rinsed with water, the bark was allowed to air-dry for three weeks in a shaded area. The bark was powdered using a Wiley mill and granulated to 80 mesh size, and three liters of 95% crude methanol were combined with 1 kg of the powdered bark to conduct an extraction. The powdered sample was subjected to maceration at room temperature for one week and subsequently filtered using Whatman No. 1 filter paper. The extraction procedure was carried out twice. Using a rotary evaporator unit (Panchun Scientific Co., Kaohsiung, Taiwan), the filtrates were combined and managed under low pressure and at 40 °C to produce a crude methanol extract (FXBM) with a final mass of 80 g. The next step included sorting the chemicals in ascending order of polarity. For this procedure, in a glass reagent bottle we dissolved 60 g of FXBM in 200 ml of dH₂O. After that, we used a sequence of increasingly polar solvents (hexane, chloroform, ethyl acetate, and H₂O) to perform a liquid-liquid partition by using a separating funnel. As the result of fractionation, 10 g of the n-hexane fraction (FXBH), 13.2 g of the chloroform fraction (FXBC), 11.8 g of the ethyl acetate fraction (FXBE), and 25 g of the remaining aqueous layer (FXBA) were obtained. Following fractionation, a rotary evaporator operating at a lower pressure was used to concentrate each fraction. After drying out, the extract and fractions were kept at 4 °C in the refrigerator till further use.

### 2.3. Gas chromatography-mass spectrometry of FXBM

The GC-Mass spectrometer equipment (Model: Thermo GC-Trace ultra-version 5.0, Thermo MS DSQ-II, Thermo Fisher, USA) and a ZB 35-MS capillary standard nonpolar column (30 m × 0.25 mm, 0.25 m film thickness) were utilized to conduct the GC-MS study. The GC-MS analysis performed in this study was intended for qualitative phytochemical profiling of FXBM. Compound identification was based on spectral matching with NIST-MS library data and published literature references. At a pace of 6°C per minute, the temperature of the oven was kept between 70°C and 260°C. The splitless injection approach was used to detect the n-hexane extract sample that was injected into the apparatus at a rate of 1 μl/min. At a rate of 1.0 ml/min, the chemicals were isolated using helium as the carrier gas [35]. When their mass spectra were compared to those from a library search (NIST-MS) and to those that were published in the literature (Chemdata.nist.gov/), the compounds found in the examined crude methanol bark extract of *F. xanthoxyloides* were identified.

### 2.4. *In vitro* anti-diabetic activity

#### 2.4.1. α-amylase enzyme inhibition.

An α-amylase inhibitory activity of *F. xanthoxyloides* extract/fractions was determined using a spectrophotometric assay with some modifications [36]. 50 µl of the sample was made in DMSO at different concentrations. Subsequently, 60 μl of 40 mM phosphate buffer (pH 6.9) was mixed with 30 μl of α-amylase enzyme (Sigma-Aldrich, USA, 9000-85-5) and incubated for 10 minutes at 37°C. After incubation, 25 μl of a 0.5% starch solution was added to each test tube. The reaction mixture was then incubated at 25°C for 10 minutes. In order to stop the reaction, 50 μl of 96 mM 3, 5-dinitrosalicylic acid (Sigma-Aldrich, USA, D0550) color reagent was added. It was placed in a boiling water bath for five minutes and then left to cool at room temperature. Acarbose (Sigma-Aldrich, USA, 56180-94-0) was used as a positive control. A sample with enzyme, substrate, and DMSO but no inhibitor was used as a negative control. The experiment was run in triplicate, and the absorbance at 405 nm was measured.

The following formula was used to get the percentage inhibition:


$$\begin{array}{c} \%\text{ Inhibition }=\;\left(\frac{\text{Negative Control absorbance }-\text{ Sample absorbance}}{\text{Negative Control absorbance}}\right)\times \text{100} \end{array}$$

Plotting the percentage of inhibition against the logarithm of the inhibitor concentration allowed for the determination of the IC_50_ values.

#### 2.4.2. α-Glucosidase enzyme inhibition.

α-glucosidase activity inhibition was performed based on the spectrophotometric assay Nair et al. with certain adjustments [37]. A solution of 10 µl of α-glucosidase (Sigma-Aldrich, USA, 9033-06-1) and 125 µl of 0.1 M phosphate buffer (pH 6.8) was added to 50 µl of sample at different concentrations, and the mixture was incubated for 20 minutes at 37ºC. The reaction was started by using 20 µl of a substrate known as 1 M 4-Nitrophenyl β-D-glucopyranoside (Sigma-Aldrich, USA, 10344-94-2) and incubated for 30 minutes. 50 µl of 0.1 N Na_2_CO_3_ was added to stop the reaction. The experiment was carried out in triplicate using acarbose as the positive control. Sample with enzyme, substrate and DMSO but no inhibitor was used as negative control. After measuring the absorbance at 405 nm, the percentage of α-glucosidase inhibition was computed as:


$$\begin{array}{c} \%\text{ Inhibition }=\;\left(\frac{\text{Negative Control absorbance }-\text{ Sample absorbance}}{\text{Negative Control Absorbance}}\right)\times \text{100} \end{array}$$

Regression analysis was used to determine the inhibitory concentration of extract or fractions needed to inhibit 50% of the activity of the enzyme (IC_50_).

### 2.5. DPP4 inhibition assay

Using the method of [38] which quantifies the quantity of free AMC (7-amino-4-methyl-coumarin) released from the DPP4 substrate, Gly-Pro-AMC, DPP4 activity was determined fluorometrically. Assays were performed in triplicate in 96-well microtiter plates using a Tecan Safire desktop fluorometer (Reading, England, UK). Fluorescence was measured at Em430 nm following illumination at Ex351 nm. Samples were made at pH = 7.4 in 50 mM HEPES buffer. Each well held 20 µl of test sample, 30 µl of 1 mM AMC substrate, and 20 µl of porcine DPP4 enzyme (EMD Millipore, UK) (1 U/ml). After one hour of moderate agitation at 37 °C, 100 µl of 3 mM acetic acid was added to the plates to halt the reactions. Final concentrations for plant extract/fractions were 1000, 500, 250, 125, 62.5, and 31.25 µg/ml. As a positive control in every experiment, berberine (IC_50_ = 13.3 µM) [39], a recently identified plant compound with DPP4 inhibitory action, was employed as a a phytochemical reference for assay validation and comparative *in vitro* evaluation and not as a clinical comparator [40]. A sample with enzyme, substrate, and DMSO but no inhibitor was used as a negative control. The concentration of plant extract or fractions needed to inhibit 50% of DPP4 activity was known as the IC_50_ value, which was calculated. The following formula was used to get the % inhibition:


$$\begin{array}{c} \%\text{ Inhibition}=\left(\frac{\text{Negative Control absorbance }-\text{ Sample absorbance}}{\text{Negative Control Absorbance}}\right)\times \text{100} \end{array}$$

### 2.6. Cell culture

The ATCC (Manassas, VA) provided the human hepatoma cell line (HepG2). In a humidified incubator at 37°C with 5% CO₂, the HepG2 cells were cultivated in DMEM (Hyclone, GE Healthcare, Pittsburgh, PA) supplemented with 10% FBS (Invitrogen, Carlsbad, CA) and 5% penicillin–streptomycin. Every cell line was kept up to date in accordance with the guidelines provided by the manufacturers.

### 2.7. Glucose uptake assay

We followed the protocol of [41] for this assay but with some modifications. After being planted in culture plates at a density of 1 × 10^5^ cells/ml, HepG2 cells were allowed to adhere and grow for 24 hours at 37°C in a humidified incubator with 5% CO₂. Prior to assaying, cells were preincubated for 48 hours at 37 °C with varying doses of FXBM, FXBH, FXBC, FXBE, and FXBA (10 μl, 125–500 μg/ml). After that, 25 μl of the incubation medium (DMEM) was added to the spent culture media, which had been diluted with 8 mM glucose, 0.1% bovine serum albumin, and phosphate-buffered saline. The mixture was then incubated for three hours at 37 °C. After that, 10 μl of the incubation media was taken out of each well and put into a fresh 96-well plate. Following the manufacturer’s instructions, the glucose assay kit (Sigma-Aldrich GAGO20, USA) was used to measure the amount of glucose in the medium. A microtiter plate reader was then used to measure the absorbance at 520 nm. The cell-containing wells were subtracted from the cell-free wells to determine the amount of glucose that the cells consumed. 5 μg/ml metformin was employed as a positive control, while untreated cells with just the incubation buffer and no test sample were utilized as the negative control. The MTT assay was used to examine the impact of *Fraxinus xanthoxyloides* extract and fractions on HepG2 cell viability [42].

### 2.8. HPLC-DAD analysis of FXBH

HPLC-DAD (Agilent 1200, Germany) equipped with a Zorbex RXC8 analytical column with a 5 μm particle size and a 25 ml capacity was used to perform high-performance liquid chromatography (HPLC-DAD) analysis of FXBH utilizing a previously reported method by Elansary et al [43]. Gallic acid was analyzed at 257 nm, catechin was examined at 279 nm, caffeic acid at 325 nm, ferulic acid was investigated at 320 nm, and quercetin was evaluated at 368 nm [32]. The column was restored for ten minutes following each run of the triplicate HPLC-DAD procedure.

### 2.9. In vivo antidiabetic study

#### 2.9.1. Animals.

We purchased 30 Sprague Dawley male rats (150–250 g) from the National Institute of Health, Islamabad, Pakistan. For acclimation, the animals were housed in polypropylene cages in the departmental animal house at room temperature and relative humidity of 44–56%. All the animals were given a regular pellet meal along with water for a period of one week. Fasting blood glucose level was checked with the help of a glucometer after an overnight fast. The study was performed according to the guidelines of the National Institute of Health, Islamabad. The protocol approval (approval no.: GCUF/ERC/2275) was granted by the Institutional Review Board (IRB No. 188), Government College University, Faisalabad, Pakistan.

#### 2.9.2. Acute toxicity studies.

The Organization for Economic Cooperation and Development’s recommendations were followed for conducting acute toxicity tests. FXBH was given orally to five male Sprague Dawley rats at several doses: 50, 100, 200, 400, 800, and 1600 mg/kg. The control group received a saline solution (10 ml/kg body weight (bw)). Salivation, sleep, lethargy, altered physical appearance, pain, pathological symptoms (purgation, diarrhea, nasal secretions, lesions, lachrymation, and piloerection), and death were all noted throughout the 14 days that these experimental rats were monitored daily. The maximum dose, 1600 mg/kg, did not result in any deaths. Lower dosages of 200 and 400 mg/kg bw were assessed in order to investigate the potential of plants to prevent diabetes [44].

***Chemical***: Sigma Aldrich Chemicals Pvt, Ltd. in Bangalore is a supplier of alloxan.

***Diabetes induction***: In this study Sprague Dawley male rats weighing 150–250 g (fasting blood glucose level of 90–110 mg/dL) were used. A single dose of intraperitoneal injection of Alloxan monohydrate (150 mg/kg) solubilized in 5% normal saline was employed for diabetes induction in overnight-fasted rats. Rats were allowed free access to 5% dextrose water to prevent hypoglycemia. After 72 hours, rats were checked for glucose level from the tail vein using a glucometer (Accu-Chek, Roche Diagnostics, Indianapolis, IND, United States). Only the rats with fasting glucose levels ≥ 250 mg/dl were considered diabetic-induced rats and were used for this study.

***Experiment Design***: The 30 animals were distributed among 5 groups containing six rats (n = 6) in each group. Diabetes was induced in all rats except those in the control group, and they were treated for 30 days following the methodology of Abdel-Barry et al. with little modification [45].

Group l was the normal control rat group, fed with vehicles only (5% DMSO).

Group II was diabetic control; all these rats were diabetic but remained untreated.

Group III was positive control, and diabetic rats were treated with glibenclamide (5 mg/kg per day).

Group IV diabetic rats were administered with FXBH (200 mg/kg) dissolved in 5% DMSO for 30 days.

Group V diabetic rats were administered with FXBH (400 mg/kg) dissolved in 5% DMSO for 30 days.

After administering a mixture of 1% chloralose and 25% urethane at a dose of 1 mg/kg for anesthesia, the animals were euthanized by decapitation. For hematological and serum analysis, blood was drawn in distinct CBC tubes and serum tubes. To extract the serum, the blood was centrifuged for 30 minutes at 4°C and 3000 rpm. After being separated, the serum was kept at -80 °C in aliquots. Ten percent phosphate-buffered formalin was used to preserve each rat’s pancreas after it was removed.

#### 2.9.4. Blood glucose level and body weight measurements.

During the course of the treatment with FXBH, the blood glucose level of rats was measured with the assistance of a glucometer, and body weight was taken on a weighing balance.

**2.9.5.Assessment of Serum Profile.**

Cholesterol level of treatment groups was measured through the protocol of Zlatkis et al. [46] while triglyceride level was estimated by following the protocol of Soloni [47]. By the addition of magnesium ions and phosphotungstic acid to the sample, low-density lipoproteins (LDL) were precipitated, and the fractions of high-density lipoprotein (HDL) were also measured by following the methods of Gidez et al. [48]. Lipase activity was determined by the pH-stat technique using tributyrin as the substrate for this protocol of Carriere et al. [49], while amylase activity was analyzed through the protocol of Cozzone et al. [50]. The enzymatic activities of ALT and AST in serum were also checked by the protocol proposed by Reitman & Frankel [51]. C-reactive protein level was also determined by following the study of Renard et al. [52]. Serum creatinine and urea levels were estimated by the diacetylmonooxime method Barker [51]. Glycosylated hemoglobin (HbA1c) was estimated from the whole blood by using HbA1c EZ 2.0 m [53].

#### 2.9.6. Histopathological study.

To carry out histological studies, the pancreas tissues from every treated group were fixed in 10% phosphate-buffered formalin solution. Following a longitudinal incision, the samples were serially dried in ethanol at 60%, 70%, 80%, and 100% before being lastly dried in xylene. After that, these preserved tissues were placed in paraffin. The implanted tissues were subsequently sliced into thin pieces that ranged in thickness from 3 to 5 µm. Cellular features were then visible after these sections were treated with hematoxylin/eosin. Bright-field microscopy (Nikon Eclipse E100LED MVR, Japan) was used to analyze the stained slices, and 40X magnification photographs were taken.

#### 2.9.7. Statistical analysis.

These results were presented in the form of mean ± standard deviations; sample size was n = 3 for *in vitro* and n = 6 for *in vivo* analyses. For the calculation of IC_50_ and for generating the graphs, GraphPad Prism 8.0.2 was utilized. Dunnet comparisons of the different treatments with +control were performed using one-way and two-way analysis of variance at *, *p* < 0.05; **, *p* < 0.01; and ***, *p* < 0.001. Statistics 8.1 was used for Tukey's HSD test to assess multiple comparisons between treatments.

## 3. Results

### 3.1. Gas chromatography-mass spectrometry of FXBH

**Fig 1:**
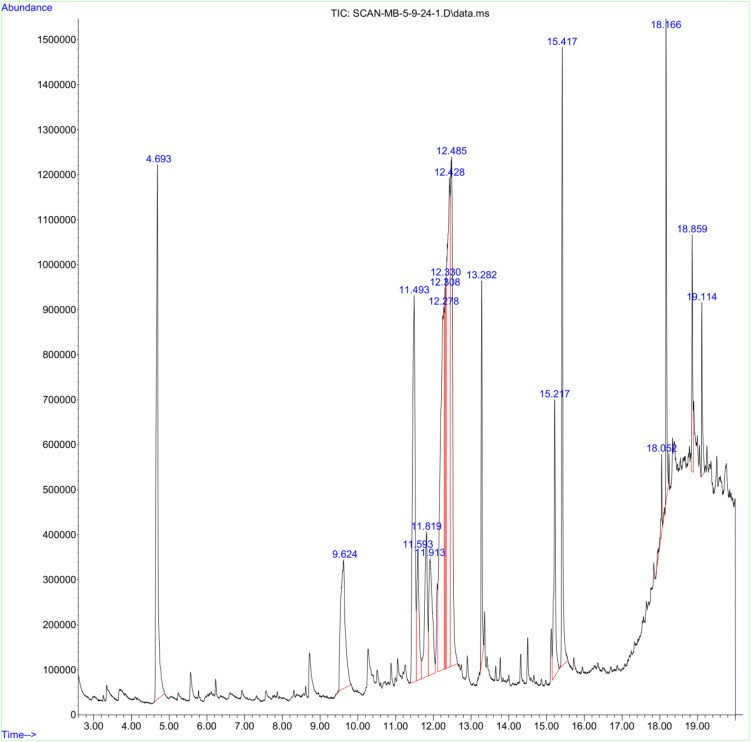
Compound identified in GC-MS analysis of FXBM.

GC-MS analysis detected a total of 17 compounds, as presented in (Table 1) in the crude methanol extract of *F. xanthoxyloides*. The chromatogram exhibited significant peaks within a retention time range of 4.69 to 19.11, as depicted in (Fig 1). The analysis revealed six major peaks, corresponding to 17 distinct compounds. These compounds were categorized as follows: four esters (17.9%), two *O*-glycosyls (12.24%), one aziridine (5.53%), two linolenic acids (10.31%), one palmitic acid (3.36%), one thiopyran (3.76%), two polyols (16.35%), one inositol (2.95%), one diazenes (10.94%), one phenol (0.65%), and one chromone (1.52%).

**Table 1:** GC-MS analysis of FXBM.

Peak number	Time (min)	Area %	Compound name	Molecular formula	MW	Compound Nature	Activity	References
1	4.693	9.28	Acrylic acid isoamyl ester	C_8_H_14_O_2_	142.2	Ester	Antioxidant and Antiinflammatory	[54]
2	9.624	5.53	1-Isopropoxy-2,2,3-trimethylaziridine (sin)	C_8_H_17_NO	143.23	Aziridines	Antitumor, Antimicrobial, and Antibacterial	[55]
3	11.493	9.40	beta-D-Glucopyranoside, methyl	C_7_H_14_O_6_	194.18	O-glycosyl	Antioxidants, Neurostimulants, and Antihypertensives	[56]
4	11.593	2.84	Ethyl.alpha.-d-glucopyranoside	C_8_H_16_O_6_	208.21	O-glycosyl		
5	11.819	3.76	D-Galactitol-5-O-hexyl-	C_12_H_26_O_6_	266.33	Polyols	---	
6	11.913	3.76	2H-Thiopyran, tetrahydro-	C_5_H_10_S	102.198	Thiopyrans	Antimicrobial and Anticonvulsant	[57]
7	12.278	12.59	D-Fructose, 3-O-methyl	C_7_H_14_O_6_	194.18	Polyols	---	
8	12.308	2.95	Myo-Inositol, 4-C-methyl	C_7_H_14_O_6_	194.18	Inositol	Neurotransmitter synthesis	[58]
9	12.330	3.15	2-[2-(2-Ethoxyethoxy) ethoxy]ethyl acetate	C_10_H_20_O_5_	220.26	Ester		
10	12.485	10.94	Diazene, butyl[1-(2,2-dimethylhydrazino)ethyl]-	C_8_H_20_N_4_	172.27	Diazenes	Antibacterial, antifungal, antiallergic, antitubercular, anti-inflammatory, analgesic, anticonvulsant, and antimalarial activities	[59]
11	13.282	3.36	n-Hexadecanoic acid	C_16_H_32_O_2_	256.42	Palmitic acid	Anticancer, antimicrobial, antiviral	[60]
12	15.217	4.02	9,12-Octadecadienoic acid (Z,Z)-	C_18_H_32_O_2_	280.44	Linoleic acid	Anticancer, anti-inflammatory, antiviral, antibacterial, neuroprotective	[61]
13	15.417	6.29	9,12,15-Octadecatrienoic acid, (Z,Z,Z)-	C_18_H_3_0O_2_	278.42	Linolenic acid		
14	18.052	0.65	Phenol, 2,2’-methylenebis[6-(1,1-dimethylethyl)-4-methyl	C_29_H_44_O_2_	424.7	Phenols	Anti-inflammatory, anticancer, anti-aging, antibacterial, and antiviral activities	[62]
15	18.166	3.54	Hexadecanoic acid, 2-hydroxy-1-(hydroxymethyl)ethyl	C_19_H_38_O_4_	330.5	Esters	Allelopathic, antimicrobial, insecticidal	[63]
16	18.859	1.93	Octadecanoic acid, 2,3-dihydroxypropyl	C_21_H_42_O_4_	358.55	Esters		
17	19.114	1.52	5H-Furo[3,2-g][1]enzopyran-5-one, 4-hydroxy-9-methoxy-7-methyl-	C_11_H_6_O_3_	186.16	Chromones	Anti-diabetes, anticancer, antioxidant, antibacterial, anticancer, and anti-inflammatory activities	[64]

### 3.2. In vitro anti-diabetic activity

**Fig 2:**
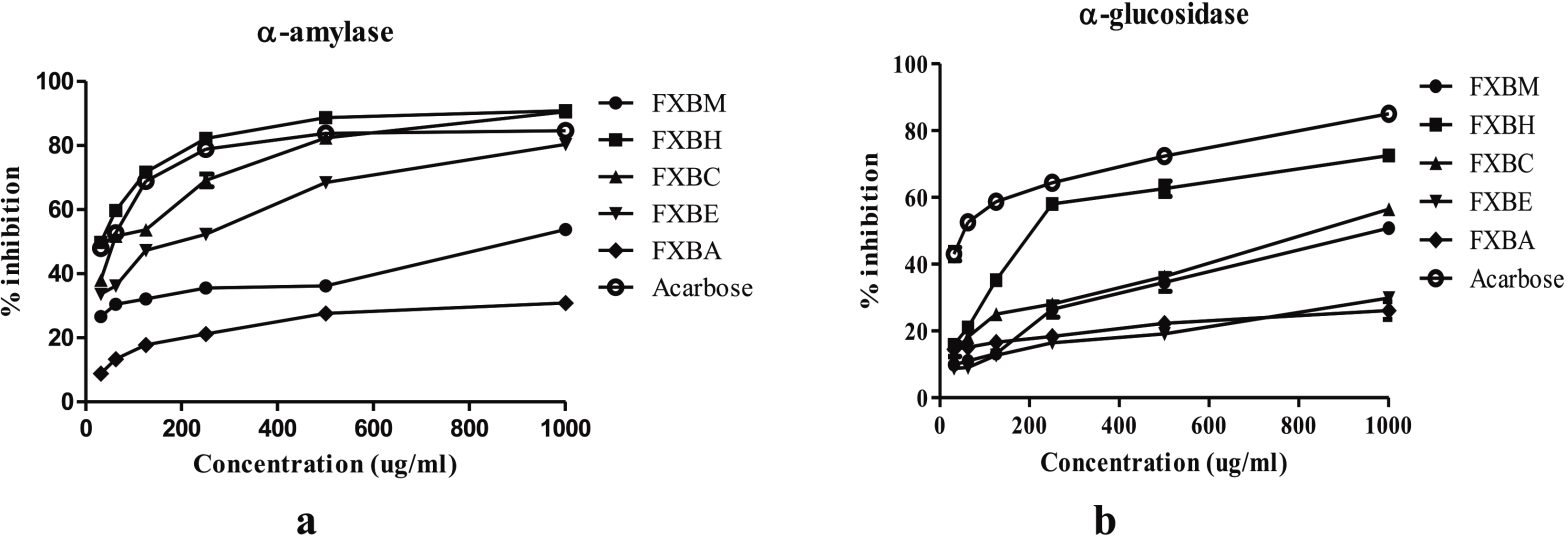
Percent Inhibitory Effect of *Fraxinus xanthoxyloides* Bark (a) α-amylase and (b) α-glucosidase. The variability test and is presented in Table 2.

All five extract/fractions of *F. xanthoxyloides* bark exhibited varying degrees of inhibition on α-amylase and α-glucosidase activity. The maximum inhibition of both enzymes was observed at a concentration of 1000 µg/ml. The assessment of α-amylase and α-glucosidase activity of the bark extract/fractions was conducted using IC_50_ standards. FXBH displayed the maximum inhibition of α-amylase (IC_50_ = 39.50 µg/ml) and α-glucosidase (IC_50_ = 245.60 µg/ml) among all extract/fractions when compared to acarbose, i.e., IC_50_ = 35.80 µg/ml for α-amylase and IC_50_ = 57.94 µg/ml for α-glucosidase as shown in (Table 2 and Fig 2). The α-amylase and α-glucosidase activity was in the following order: FXBH < FXBC < FXBE < FXBM < FXBA and FXBH < FXBC < FXBM < FXBA < FXBE, respectively.

**Table 2:** IC_50_ values of *Fraxinus xanthoxyloides* bark against α-amylase, α-glucosidase, and DPP4 activity.

Sample	Concentration µg/ml	α-Amylase		α-Glucosidase		DPP4	
		Mean±SD	IC_50_ (µg/ml)	Mean±SD	IC_50_ (µg/ml)	Mean±SD	IC_50_ (µg/ml)
Acarbose Berberine	1000	84.64 ± 1.16	39.50	85.00 ± 0.03	57.94	87.72 ± 1.72	19.17
	500	83.78 ± 1.03		72.33 ± 0.03		79.82 ± 1.32	
	250	78.89 ± 0.76		64.33 ± 1.50		73.07 ± 0.47	
	125	68.85 ± 1.08		58.67 ± 1.60		69.17 ± 0.64	
	62.5	52.70 ± 1.31		52.50 ± 1.45		62.11 ± 0.44	
	31.25	47.93 ± 1.64		43.00 ± 0.34		56.49 ± 1.03	
FXBM	1000	53.80 ± 1.36^***^	1392	50.76 ± 1.72^***^	1036	50.32 ± 0.59^***^	894.3
	500	36.17 ± 2.00^***^		34.56 ± 4.60^***^		43.37 ± 2.15^***^	
	250	35.56 ± 1.09^***^		26.43 ± 3.94^***^		33.84 ± 2.30^***^	
	125	32.13 ± 1.49^***^		13.27 ± 2.19^***^		24.54 ± 2.71^***^	
	62.5	30.40 ± 1.10^***^		11.00 ± 1.65^***^		19.43 ± 0.98^***^	
	31.25	26.60 ± 2.30^***^		9.90 ± 1.41^***^		12.73 ± 1.19^***^	
FXBH	1000	90.83 ± 1.70^**^	33.38	72.5 ± 3.15^***^	245.60	73.98 ± 2.47^***^	195.8
	500	88.70 ± 2.35^*^		62.60 ± 3.97^***^		65.92 ± 1.10^***^	
	250	82.23 ± 1.66 ^ns^		58.03 ± 2.93 ^ns^		54.72 ± 2.05^***^	
	125	71.70 ± 2.62 ^ns^		35.23 ± 2.10^***^		43.53 ± 1.26^***^	
	62.5	59.70 ± 3.05^***^		20.03 ± 2.10^***^		31.79 ± 2.17^***^	
	31.25	49.70 ± 2.92 ^ns^		15.90 ± 2.22^***^		20.10 ± 1.65^***^	
FXBC	1000	90.56 ± 2.27^**^	68.95	56.43 ± 2.13^***^	907.80	70.29 ± 1.83^***^	249.4
	500	82.40 ± 3.10 ^ns^		36.27 ± 2.68^***^		63.07 ± 2.79^***^	
	250	69.13 ± 3.45^***^		28.00 ± 2.76^***^		50.65 ± 2.47^***^	
	125	53.70 ± 2.38^***^		25.00 ± 2.65^***^		39.27 ± 2.82^***^	
	62.5	51.66 ± 3.17 ^ns^		18.20 ± 2.10^***^		28.10 ± 4.01^***^	
	31.25	38.00 ± 1.68^***^		13.60 ± 2.13^***^		16.70 ± 0.97^***^	
FXBE	1000	80.40 ± 2.26 ^ns^	143.60	29.90 ± 2.01^***^	6520	57.22 ± 1.74^***^	571.2
	500	68.46 ± 2.05^***^		20.93 ± 2.95^***^		47.68 ± 2.01^***^	
	250	52.33 ± 2.15^***^		16.43 ± 1.06^***^		38.58 ± 2.61^***^	
	125	47.20 ± 2.30^***^		12.66 ± 2.17^***^		29.82 ± 3.20^***^	
	62.5	36.2 ± 1.51^***^		9.00 ± 2.33^***^		20.63 ± 2.69^***^	
	31.25	33.60 ± 2.80^***^		8.76 ± 1.72^***^		8.71 ± 2.77^***^	
FXBA	1000	30.90 ± 2.02^***^	6321	22.06 ± 2.90^***^	115802	50.74 ± 0.99^***^	936.5
	500	27.60 ± 1.99^***^		20.43 ± 2.31^***^		43.29 ± 1.97^***^	
	250	21.23 ± 1.81^***^		18.00 ± 1.49^***^		36.23 ± 1.62^***^	
	125	17.80 ± 1.57^***^		17.16 ± 1.55^***^		29.55 ± 2.14^***^	
	62.5	13.37 ± 2.23^***^		16.10 ± 1.60^***^		24.32 ± 2.69^***^	
	31.25	8.86 ± 1.12^***^		15.70 ± 1.70^***^		18.75 ± 1.90^***^	

Data values are expressed as mean ± SD (n = 6). For the Dunnet comparison of treatments with the (+)-control, two-way analysis of variance (ANOVA) was followed at *p < 0.05, **p < 0.001, ***p < 0.0001, and ^ns^ non-significant.

### 3.3. DPP4 inhibition assay

**Fig 3:**
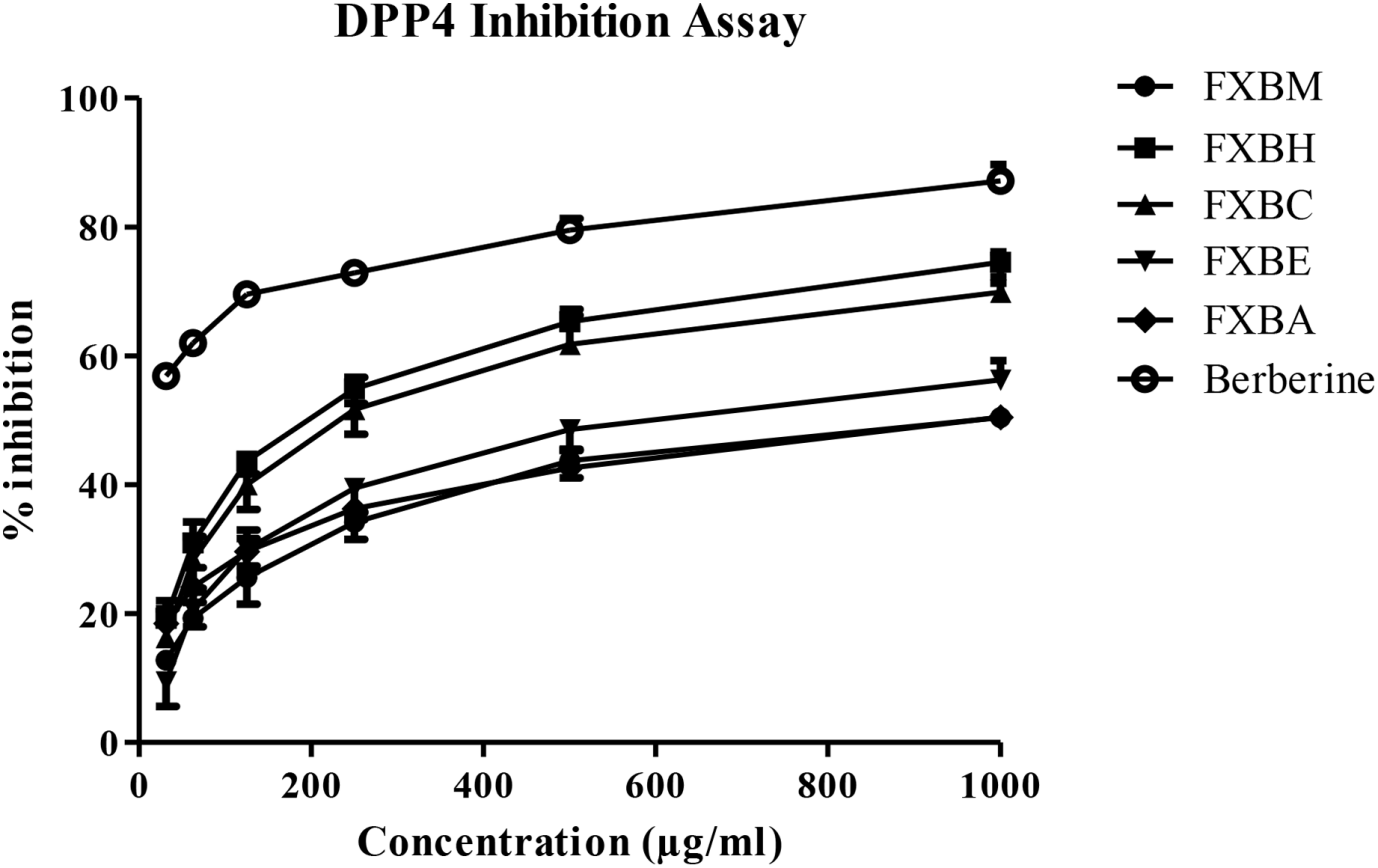
Percent inhibition of *Fraxinus xanthoxyloides* bark extract and its fractions against DPP4 activity. Variability data is presented in Table 2.

All the extract/fractions of *F. xanthoxyloides* bark exhibited varying degrees of inhibition on DPP4 enzyme activity. The maximum inhibition of DPP4 was observed at a higher concentration (1000 µg/ml), and there was an increase in percentage inhibition with the increase in concentration. FXBH displayed the maximum inhibition of DPP4 (IC_50_ = 195.80 µg/ml) when compared with Berberine (IC_50_ = 19.17 µg/ml). The DPP4 inhibitory activity was in the following order: FXBH < FXBC < FXBE < FXBM < FXBA (Table 2 and Fig 3).

### 3.4. MTT assay

**Fig 4:**
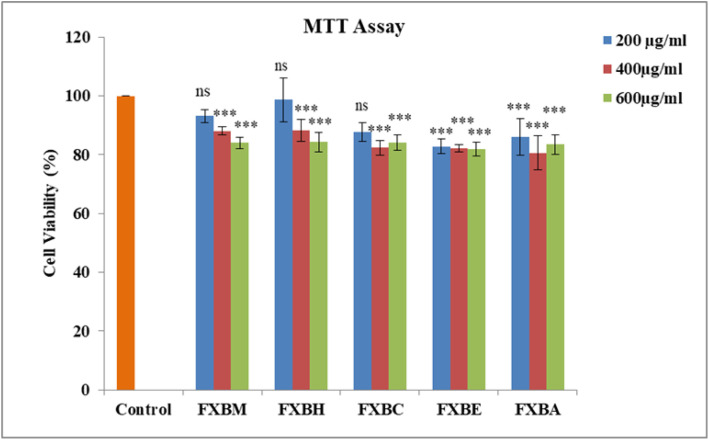
Effect of *Fraxinus xanthoxyloides* bark extract/fractions on cell viability in HepG2 hepatocytes. Data are expressed as mean ± SD (n = 3). For Dunnet comparison of treatments with the untreated control, one-way analysis of variance (ANOVA) was performed at ***p < 0.001 and ^ns^ non-significant.

Using the MTT assay, the extract/fractions’ cytotoxic activity against the HepG2 liver cell line was assessed at several concentrations, including 200 µg/ml, 400 µg/ml, and 600 µg/ml. The results of the cytotoxicity assay showed that *Fraxinus xanthoxyloides* extract/fractions displayed a low level of toxicity to HepG2 cells. Thus, all the extract/fractions showed >80% cell viability at all the doses tested in a dose-dependent manner (Fig 4).

### 3.5. Glucose uptake assay

**Fig 5:**
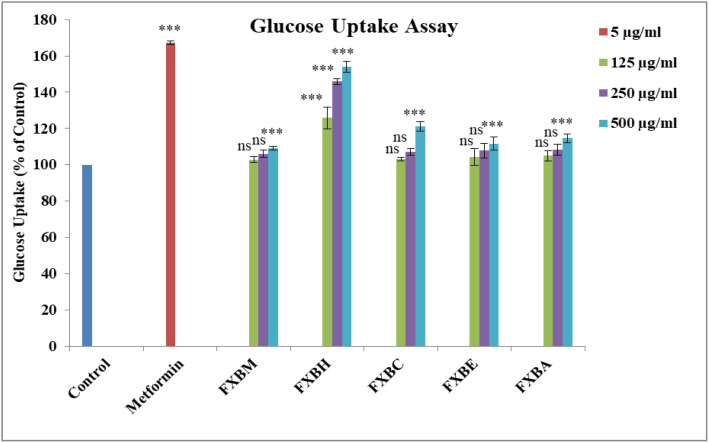
Effect of *Fraxinus xanthoxyloides* bark extract/fractions on glucose utilization in HepG2 hepatocytes. Data expressed as mean ± SD (n = 3). For Dunnet comparison of treatments with the untreated control, one-way analysis of variance (ANOVA) was performed at ***p < 0.001 and ^ns^ non-significant.

Fig 5 displays the glucose uptake findings in HepG2 cells when the plant extract or fractions are present. At all doses, the FXBH demonstrated a considerably (p < 0.001) greater increase in glucose absorption in HepG2 cells. In a concentration-dependent manner, it was 125.90%, 145.90%, and 154.05% at 125 µg/ml, 250 µg/ml, and 500 µg/ml, respectively, in comparison to the untreated control. However, when compared to the control, the glucose absorption of the FXBM, FXBC, FXBE, and FXBA was significantly higher (*p* < 0.001) only at higher concentrations (500 µg/ml), but it was still lower than that of metformin (167.27%).

### 3.6. HPLC-DAD analysis of FXBH

**Fig 6:**
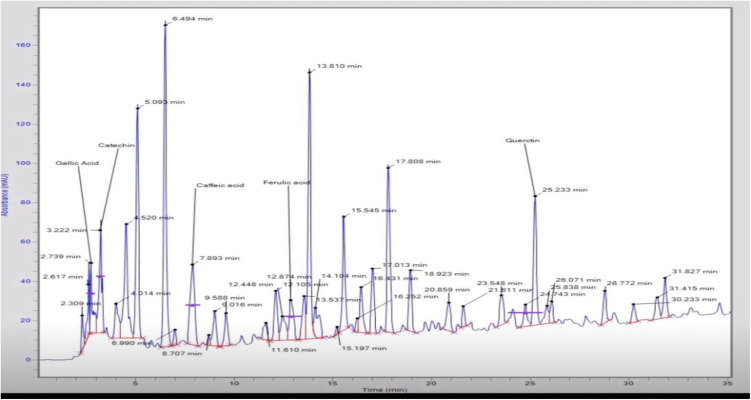
HPLC-DAD chromatogram of FXBH fraction.

The chromatogram of FXBH revealed the presence of several prominent peaks at various retention times. Peak 3 (RT: 2.739 min) was identified as gallic acid (3.29 µg/mg), peak 4 (RT: 3.222 min) was recognized as catechin (4.23 µg/mg), peak 10 (RT: 7.893 min) was confirmed to be caffeic acid (6.05 µg/mg), and peak 17 (RT: 12.874 min) was determined to be ferulic acid (2.99 µg/mg). Peak 32 displayed the maximum retention time (RT = 25.233 min) and was designated as quercetin (6.40 µg/mg) (Table 3 and Fig 6).

**Table 3:** HPLC-DAD profile of FXBH fraction.

Peak	RT (min)	Component Name	Area	Height	Concentration µg/ml
3	2.739	Gallic acid	329135.2	36699.9	3.29
4	3.222	Catechin	507231.9	46311.9	4.23
10	7.893	Caffeic acid	665572.2	41241.5	6.05
17	12.874	Ferulic acid	314360.2	20525.3	2.99
32	25.233	Quercetin	694345.1	64889.7	6.40

### 3.7. In vivo antidiabetic assay

#### 3.7.1. Effect of FXBH on body weight (g).

**Fig 7:**
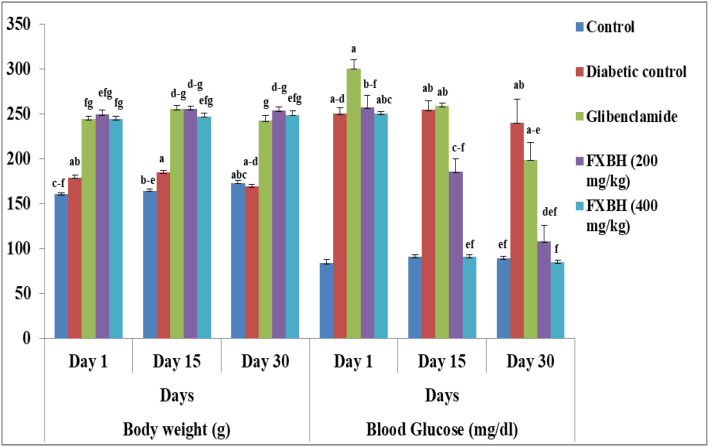
Effect of FXBH on body weight (g) and blood glucose (mg/dl) of rats. Data values are expressed as Mean ± SD (n = 6). Different superscripts (a-g) in each column indicate differences at the *p* < 0.05 level. The Tukeys’ HSD test was used to assess multiple comparisons between treatments.

The impact of FXBH extract on body weight was assessed on the 1^st^, 15^th^, and 30^th^ days and compared with both the control and diabetic control groups, as depicted in (Fig 7). The average body weight of rats treated with FXBH at a dose of 400 mg/kg exhibited a gradual increase from day 1 to day 15 and subsequently from day 15 to day 30, with values of 244.20 ± 3.29 g, 247.40 ± 3.88 g, and 249.00 ± 4.42 g, respectively. In contrast, the body weight of the diabetic group decreased from 179.40 ± 2.94 g to 169.40 ± 2.66 g.

#### 3.7.2. Effect of FXBH on fasting blood glucose level.

The fasting blood glucose level was significantly higher in alloxan-induced diabetic rats than in the normal control group (p < 0.05). Nevertheless, fasting blood glucose levels were successfully lowered and brought closer to those of the control group by administering two distinct dosages of FXBH (200 and 400 mg/kg body weight). As shown in Fig 7, a substantial drop in blood glucose levels during the 30-day treatment was noted (p < 0.05) with the various doses of FXBH as compared to the control group and the glibenclamide standard group.

### 3.8. Effect of FXBH on lipid profile and amylase level

Lipid profile findings revealed a significant increase in the levels of cholesterol, triglycerides, low-density lipoproteins (LDL), lipase, and amylase in the diabetic group when compared with the control group, while the diabetic group exhibits a marked decrease in high-density lipoproteins (HDL).

It was clear from the value of the probability (*p* < 0.05) that there were highly significant variations between groups. In the control group, cholesterol, triglycerides, LDL, lipase, and amylase levels in healthy rats were determined to be 153.80 ± 4.43 mg/dl, 102.60 ± 4.03 mg/dl, 52.40 ± 3.43 mg/dl, 191.60 ± 2.40 mg/dl, and 206.40 ± 1.67 mg/dl, respectively, but it was found that the lipid profile as well as the level of amylase increased significantly (p < 0.05) to 201.00 ± 5.08 mg/dl (cholesterol), 231.40 ± 3.20 mg/dl (triglycerides), 122.80 ± 3.69 mg/dl (LDL), 404.40 ± 2.96 mg/dl (lipase), and 977.20 ± 7.66 mg/dl (amylase), respectively, in the diabetic control group. While high-density lipoprotein (HDL) levels were recorded to be 65.60 ± 3.36 mg/dl in the control group, a considerable reduction was noted (42.20 ± 2.58 mg/dl) in the diabetic group. Lipid profile and amylase level in all groups exhibited significant variations from the control group.

Consequently, the administration of two different doses (200 and 400 mg/kg) of FXBH resulted in a notable (*p* < 0.05) reduction in lipid profile and amylase level. It was observed that a high dose (400 mg/kg) of FXBH was more significant in lowering cholesterol (169.00 ± 2.23 mg/dl), triglycerides (120.40 ± 2.70 mg/dl), LDL (44.60 ± 3.43 mg/dl), lipase (209.00 ± 2.73 mg/dl), and also the amylase level (444.20 ± 3.49 mg/dl), while simultaneously elevating the HDL level to approach that of the control group. FXBH (400 mg/kg) increased the high-density lipoproteins (HDL) to 89.54 ± 6.17 mg/dL. The groups treated with glibenclamide also exhibited a statistically significant improvement in these values when compared to the diabetic control group, as shown in (Table 4).

**Table 4:** Effect of FXBH on lipid profile and amylase level in rats.

Group	Cholesterol (mg/dl)	Triglyceride (mg/dl)	LDL (mg/dl)	HDL (mg/dl)	Lipase level (mg/dl)	Amylase level (mg/dl)
Control	153.80 ± 4.43^ab^	102.60 ± 4.03^e^	52.40 ± 3.43^bc^	65.60 ± 3.36^a^	191.60 ± 2.40^d^	206.40 ± 1.67^e^
Diabetic control	201.00 ± 5.08^a^*	231.40 ± 3.20^a^*	122.80 ± 3.69^a^*	42.20 ± 2.58^b^ *	404.40 ± 2.96^a^*	977.20 ± 7.66^b^*
Glibenclamide	142.00 ± 2.73^b^	139.60 ± 5.59^c^*	60.60 ± 3.91^bc^	55.40 ± 2.60^c^*	276.60 ± 1.51^b^*	561.00 ± 4.30^b^*
FXBH (200 mg/kg)	170.40 ± 3.57^ab^	147.60 ± 3.97^b^*	85.60 ± 15.17^b^*	72.89 ± 5.64^b^	205.60 ± 4.98^c^*	493.00 ± 4.18^d^*
FXBH (400 mg/kg)	169.00 ± 2.23^ab^	120.40 ± 2.70^d^*	44.60 ± 3.43^c^	89.54 + 6.17^d^*	209.00 ± 2.73^c^*	444.20 ± 3.49^c^*

Data values are expressed as mean ± SD (n = 6). Different superscripts (a-e) in each column indicate a difference at the *p* < 0.05 level. The Tukeys’ HSD test was used to assess multiple comparisons between treatments. For the Dunnet comparison of treatments with the control, one-way analysis of variance (ANOVA) was performed at *p < 0.05.

### 3.9. Effect of FXBH on serum markers and HBAIC level

The optimal ALT level in healthy rats was established at 38.30 ± 2.49 mg/dl, but it increased significantly (*p* < 0.05) to 111.00 ± 4.94 mg/dl in the diabetic control group. Nevertheless, this heightened ALT level was substantially diminished in the group that received a 200 mg/kg FXBH treatment (43.20 ± 1.92 mg/dl) when compared with the rats treated with glibenclamide and the control group. The normal AST level in healthy rats was determined to be 41.80 ± 3.34 mg/dl, but it significantly increased (*p* < 0.05) to 218.00 ± 2.42 mg/dl in the diabetic control group. However, this elevated AST level was notably reduced in the group treated with 400 mg/kg FXBH (50.80 ± 2.77 mg/dl) when compared to the rats treated with glibenclamide, where AST levels were observed at 125.20 ± 3.34 mg/dl. This observation suggests potential liver damage, as indicated in (Table 5). The test for C-reactive protein levels indicates a prognostic marker for pancreatic necrosis, displaying the highest sensitivity and negative prognostic value, with a cut-off of 110 mg/l. The C-reactive protein level in the diabetic group increased significantly (p < 0.05) to 33.44 ± 0.48 mg/dl in comparison to the control (10.10 ± 0.43 mg/dl) group. The findings revealed that the administration of 200 and 400 mg/kg of FXBH resulted in lowering the level of C-reactive protein, i.e., 10.00 ± 0.72 mg/dl and 11.92 ± 1.37 mg/dl, respectively, which was comparable to the normal control group. Similarly, creatinine (1.35 ± 0.11 mg/dl) and urea (21.84 ± 0.89 mg/dl) levels in diabetic rats increased significantly (p < 0.05) in comparison to the control group, suggesting diabetes-induced damage in the liver and, to some extent, also in the kidney, which may have disrupted the normal urea cycle in the liver. After the administration of 200 mg/kg of FXBH, the level of creatinine was 0.22 ± 0.06 mg/dl and urea was 11.64 ± 0.47 mg/dl, and also 400 mg/kg of FXBH helped to reduce the creatinine (0.62 ± 0.02 mg/dl) and urea (11.44 ± 0.42 mg/dl) levels, bringing them closer to those observed in the control group as shown in (Table 5).

**Table 5:** Effect of FXBH on serum markers and HbA1c level.

Group	ALT (mg/dl)	AST level (mg/dl)	C-reactive protein level (mg/dl)	Creatinine (mg/dl)	Urea (mg/dl)	HbA1c (mg/dl)
Control	38.30 ± 2.49^c^	41.80 ± 3.34^c^	10.10 ± 0.43^c^	0.46 ± 0.07^c^	11.20 ± 0.8^c^	5.58 ± 0.13^bc^
Diabetic control	111.00 ± 4.94^a^*	218.00 ± 2.42^a^*	33.44 ± 0.48^a^*	1.35 ± 0.11^a^*	21.84 ± 0.89^a^*	9.68 ± 0.19^a^*
Glibenclamide	45.60 ± 4.39^b^*	125.20 ± 3.34^b^*	11.56 ± 1.12^bc^	0.58 ± 0.084^bc^	13.48 ± 0.54^b^*	6.20 ± 0.48^b^*
FXBH (200 mg/kg)	43.20 ± 1.92^bc^	52.40 ± 3.04^c^*	10.00 ± 0.72^c^	0.22 ± 0.06^d^*	11.64 ± 0.47^c^	6.14 ± 0.42^b^*
FXBH (400 mg/kg)	45.60 ± 1.81^b^*	50.80 ± 2.77^c^	11.92 ± 1.37^b^*	0.62 ± 0.02^b^*	11.44 ± 0.42^c^	5.10 ± 0.29^c^

Data values are expressed as mean ± SD (n = 6). Different superscripts (a–d) in each column indicate differences at the p < 0.05 level. The Tukeys’ HSD test was used to assess multiple comparisons between treatments. For the Dunnet comparison of treatments with the control, one-way analysis of variance (ANOVA) was performed at *p < 0.05.

Blood analysis evaluates the amount of glucose associated to hemoglobin, and the findings suggested that diabetic rats exhibited a high level of HbA1c (9.68 ± 0.19 mg/dl) in their blood compared to the control group (5.58 ± 0.13 mg/dl). HbA1c levels in diabetic rats administered with FXBH were considerably lower than in the diabetic control group, but a high dose (400 mg/kg) of FXBH led to a substantial reduction in HbA1c to a 5.10 ± 0.29 mg/dl level, bringing them in line with those of the control group as indicated in (Table 5).

### 3.10. Histopathological analysis

**Fig 8:**
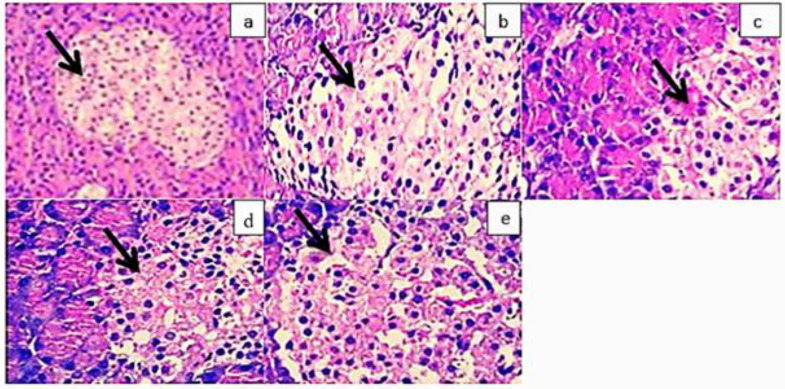
Histopathological studies of the pancreas. (a) Control group (b) Diabetic control (c) Pretreated with Alloxan (150 mg/kg) + Glibenclamide (5 mg/kg) (d) Pretreated with Alloxan (150 mg/kg) + FXBH (200 mg/kg) (e) Pretreated with Alloxan (150 mg/kg) + FXBH (400 mg/kg).

A normal rat pancreas showed typical islets of Langerhans with pale, rounded, and ovoid cells in the center (Fig 8a). In the pancreas of diabetic rats, shrinkage of the islets of Langerhans was observed along with the degeneration and necrosis of the cells within them. This is evident from the highly basophilic nuclei and the presence of karyolysis (Fig 8b). Diabetic rats treated with glibenclamide revealed typical islets of Langerhans with their usual pale, big, round to ovoid cells implanted in the exocrine pancreas (Fig 8c). Diabetic rats treated with FXBH showed normal-sized islets of Langerhans, although there was some degradation of the cell in the middle of the pancreas (Fig 8d & e).

## 4. Discussion

Hypoglycemic medicines possess serious adverse health effects, and these are also expensive for the majority of individuals. Medicinal plants are a key source of pharmaceuticals because compounds extracted from plants play an important part in the drug discovery approach [65].

This is an early and comprehensive study to assess the antidiabetic effects of *Fraxinus xanthoxyloides* bark extract in rats with diabetes induced by alloxan and bark extract from *F. xanthoxyloides* may be a useful medication option because of its possible antidiabetic effects. The numerous applications of *F. xanthoxyloides* leaf and bark extract/fractions in biological systems have been shown by our multiple prior in vitro and in vivo investigations. Since ancient times, *F. xanthoxyloides* bark extract has been widely used in traditional folk treatments [66].

The phytochemical profile generated by GC-MS analysis identified that FXBM contains 17 different compounds with six major peaks, as detailed in Table 1. It is important to note that the GC-MS analysis conducted in this study was qualitative in nature and aimed at profiling volatile and semi-volatile constituents of FXBM. Although relative peak area percentages are reported, no quantitative calibration using reference standards was performed. The identification of compounds relied on spectral matching with NIST-MS library data and literature comparisons. Fig 1 shows the retention periods for the different components. These compounds were categorized as follows: four esters (17.9%), two O-glycosyl (12.24%), one aziridine (5.53%), two linolenic acids (10.31%), one palmitic acid (3.36%), one thiopyran (3.76%), two polyols (16.35%), one inositol (2.95%), one diazenes (10.94%), one phenol (0.65%), and one chromone (1.52%) (Table 1). Fatty acids and phenols may be responsible for the extract’s or fractions’ anti-diabetic properties [67]. The various chemical combinations and amounts in the corresponding extracts or fractions may be the cause of the variation in extract/fraction activity seen in this investigation. Among the phyto-constituents discovered by GC-MS analysis, ethyl α-D-glucopyranoside is a naturally occurring glycoside that activates the glycogenesis route by targeting the PI3K-GSK-3 and MAPK-PP1 pathways. It has demonstrated anti-diabetic activities by enhancing glucose uptake and promoting upregulation in the glycogenesis pathway [68].

The *in vitro* antidiabetic potential of *F. xanthoxyloides* bark extract and fractions is investigated in this work. HPLC and *in vivo* antidiabetic profiling are used to further examine the extract or fraction exhibiting the most potent antidiabetic activity. The inhibitory properties of *F. xanthoxyloides* bark were investigated using two *in vitro* procedures. Acarbose, a complex oligosaccharide that inhibits many enzymes, was once a common antidiabetic medication. It prevents the breakdown of complex starches, oligosaccharides, trisaccharides, and disaccharides into absorbable simple sugars by inhibiting both membrane-bound α-glucosidases and pancreatic α-amylase [69]. The present study used IC_50_ to evaluate the in vitro effects of *F. xanthoxyloides* bark extract/fractions on α-amylase and α-glucosidase inhibition. FXBH showed the highest activity of α-amylase (IC_50_ = 39.50 µg/ml) and α-glucosidase (IC_50_ = 245.60 µg/ml) in comparison to acarbose (IC_50_ = 35.80 µg/ml and IC_50_ = 57.94 µg/ml, respectively) (Table 2 and Fig 2).

The findings of our research are in accordance to Perez Gutierrez et al. [70] whose research described the anti-diabetic properties of *Piper auritum* in streptozotocin (STZ)-induced diabetic rats at a dosage of 60 mg/kg. The results of this study demonstrated that a concentration of 250 g/ml of the compound, characterized from the extract of *Piper auritum*, inhibited α-amylase and α-glucosidase by 76.61% and 65.08%, respectively. Additionally, this supports earlier findings by Karakaya et al. [71] that the root extract of *Ferulago bracteata* has α-amylase and α-glucosidase inhibitory qualities. In contrast to the reference standard, acarbose, which had an IC_50_ of 4.95 mg/ml, the study results showed that elamidin and suberosin had notable α-glucosidase inhibitory effects, with IC_50_ values of 0.42 mg/ml and 0.89 mg/ml, respectively.

FXBH exhibited the strongest DPP4 inhibitory activity with an IC_50_ of 195.80 µg/ml as shown in (Table 2 and Fig 3) when compared to that of Berberine with an IC_50_ of 19.17 µg/ml, and the order of DPP4 inhibitory activity among the extract was FXBH < FXBC < FXBE < FXBM < FXBA. The study by Quek et al. [67] reported that the bark extract of *M. latifolia* has the strongest DPP4 inhibitory activity, with the identification of methyl p-coumarate as the compound responsible, exhibiting the highest DPP4 inhibitory activity with an IC_50_ of 911.44 μM.

The cytotoxicity results indicated that the *F. xanthoxyloides* extract and its fractions demonstrated low toxicity towards HepG2 cells, with all doses tested showing greater than 80% cell viability in a dose-dependent manner as shown in (Fig 4). This aligns with previous findings by Sagbo et al. [72] who reported that the leaf extract of *Brachylaena elliptica* exhibited a low level of toxicity, as neither of the tested concentrations induced 50% cell death. FXBH significantly (p < 0.001) enhanced glucose uptake in HepG2 cells in a concentration-dependent manner, with increases of 125.90%, 145.90%, and 154.05% at concentrations of 125 µg/ml, 250 µg/ml, and 500 µg/ml, respectively, compared to the untreated control as shown in (Fig 5). This is consistent with earlier research by Beidokhti et al. [73], which showed that *Psidium guajava* L. leaves increased glucose uptake by approximately 161% in C2C12 muscle cells compared to the dimethyl sulfoxide (DMSO) control, similar to the effects of the reference compounds metformin (144.0%) and insulin (141.5%).

After conducting in *vitro* assays, we concluded that FXBH had more antidiabetic potential as compared to other extract/fractions, so we made our choice to move further with this fraction. HPLC was used to further analyze FXBH, and the chromatographic outline was compared to the absorption spectrum and retention durations of the reference standards (quercetin, gallic acid, catechin, caffeine, and ferulic acid). The HPLC profile showed that the compound that comprises the maximum retention time (RT = 25.233 min) at Peak 32 and thus higher concentration (6.40 µg/mg) was designated as quercetin (Fig 6). According to Ansari et al. [74], quercetin has demonstrated antidiabetic properties while improving glucose tolerance and metabolism. Further, *in* *silico* studies sustained the potential inhibitory role of quercetin against α-amylase *via* hydrogen bonds (active site Asp197 and Gln63) and hydrophobic interactions (with active site residues Ala198, Leu165, and Trp59) in a mixed-competitive manner [75]. Also, quercetin demonstrated a reversible, slow-binding, and non-competitive inhibition of α-glucosidase (K_i_ value = 6.3 × 10^−8^ M) [76]. Moreover, quercetin showed strong inhibitory binding activity against DPP4 with IC_50_ value of 4.02 µM [77]. Likewise, Narasimhan et al. [78] proved that ferulic acid lowers gluconeogenesis and insulin signaling negative regulators while notably raising hepatic glycogenesis and insulin sensitivity in type 2 diabetic rats. Using enzyme kinetic analysis, ferulic acid showed remarkable α-amylase and α-glucosidase inhibitory effects with IC_50_ values of 0.622 and 0.866 mg/ml *via* mixed and non-competitive mechanisms [79].

Moreover, Xu et al. [80] studied that in spite of reducing blood glucose, caffeic acid additionally improves serum insulin levels. Another study has proved the inhibitory effect of caffeic acid against α-amylase (IC_50_ = 3.68 μg/ml) and α-glucosidase (IC_50_ = 4.98 μg/ml) (Oboh et al. 2015). According to Samarghandian et al. [81], catechin can potentially mitigate diabetes and its complications by regulating oxidative stress. Furthermore, in a gallic acid-mediated model, gallic acid can suppress glucose levels by directly targeting the overexpression of pAkt, PPAR-γ, and Glut4 receptors [82]. A certain study has proved that the combination of gallic acid (50%) and acarbose (50%) showed the highest α-glucosidase and α-amylase inhibitory effect, compared with acarbose alone [83]. The molecular docking studies of gallic acid showed competitive binding to the active site of α-amylase in a 1:1 stoichiometry, dominantly *via* four hydrogen bonds and one hydrophobic interaction [84]. Additionally, gallic acid obtained from *Punica granatum* peel extracts presented DDP4 inhibitory activity [85].

Moreover, the current study also evaluated *F. xanthoxyloides'* ability to ward off diabetes that has been triggered by alloxan in rats. It has been noted that reactive radical species from alloxan caused destruction to the pancreas. Treatment with FXBH and glibenclamide increased the glucose metabolism, and so before it, a decrease in body weight in affected rats was observed. It has been found that treatment with alloxan causes a decrease in body weight as compared to the control group. The average body weight of rats treated with FXBH (400 mg/kg) showed a gradual increase in weight from day 1 to 15 and then from day 15 to 30 (Fig 7). The findings of our study are in accordance with those previously reported by Provilus et al. [86], which show a direct correlation between the increase in body weight and the administration of antidiabetic agents. Different concentrations of FXBH, as well as glibenclamide, significantly lowered blood glucose levels from day 1 to day 30. The highest reduction in blood glucose level was observed with the administration of 400 mg/kg of FXBH to diabetic rats (Fig 7). In a similar context, Gupta et al. [87] demonstrated that methanolic extract (200 mg/kg) of *Passiflora incarnata* significantly reduced the blood glucose levels in streptozotocin-induced diabetic mice.

The presence of cholesterol carrying LDL is linked to the advancement of atherosclerosis and the narrowing or blockage of the arterial lumen. LDL plays a crucial role in the transport of lipids even in the smallest capillaries. The present study revealed that diabetic rats exhibited a minimal HDL level. However, the administration of FXBH at dosages of 200 mg/kg and 400 mg/kg resulted in a significant increase in HDL levels alongside decreasing LDL levels. Lipase is a crucial metabolic enzyme responsible for regulating the lipid profile. In this study, a maximum reduction in lipase level was achieved with a low dosage of FXBH (Table 4). In a similar fashion, Nabi et al. [88] observed that polyherbal combinations with antidiabetic effects increase the serum HDL levels in streptozotocin-induced diabetic rats. The amylase level has been reported to be a maximum in Sprague Dawley rats being administered with alloxan. When α-amylase does not stop its function, then an excess conversion of starch to sugar has been noted, and as a result, the sugar level increased in the blood. Different concentrations of FXBH decreased the serum level of amylase (Table 4). These are in agreement with Udia et al. [89] whose study reported that an extract of *Rothmannia hispida* significantly possessed the ability to lower the amylase concentration.

Elevated ALT levels in the blood serum have been linked to liver injury in individuals with diabetes. However, in this study, FXBH at a dose of 200 mg/kg reduced the ALT levels to 43.20 mg/dl, a result comparable to the high dose of FXBH. Furthermore, FXBH at 400 mg/kg decreased ALT levels to 45.60 mg/dl. These results are in agreement with those obtained in a previous study by Nabi et al. [88] which explained the antidiabetic effect of methanolic extract of *Piper longum* root extract in diabetic rats. The administration of 200 mg/kg of aqueous extract from *Piper longum* root significantly reduced the markers of liver and kidney function. Elevated AST levels in diabetic rats suggested active liver injury. The rise in serum AST and ALT levels indicates that diabetes primarily results from enzyme leakage into the bloodstream from liver cytosol, revealing the hepatotoxic effects of alloxan. Diabetic rats exhibited a significantly elevated level of AST as compared to the control group. Glibenclamide also reduced AST levels in comparison to the diabetic group. FXBH at 200 mg/kg lowered the AST levels, while FXBH at 400 mg/kg further decreased AST levels. A high dose of FXBH showed a maximum decrease in serum level of AST as compared to glibenclamide (Table 5). Our findings were in the context of Ahmed et al. [90], which shows AST and ALT levels came to a normal range due to flavonoids' presence in plant extract. Diabetic rats exhibit higher CRP concentrations in their blood serum compared to normal rats, which can lead to cardiovascular diseases. FXBH 200 mg/kg decreased the CRP level more as compared to FXBH 400 mg/kg. This showed that high and low doses of FXBH have a considerable effect on declining the serum level of CRP (Table 5). Our findings are in relation to Sadjadi et al. [91] where a short-term caraway extract administration decreased the cardiovascular disease risk markers in streptozotocin-induced diabetic rats, which is due to a decrease in CRP level.

In this study, FXBH 200 mg/kg decreased the higher creatinine level, while FXBH 400 mg/kg decreased the creatinine level, which was lower in value as compared to FXBH 200 mg/kg. In the same way, Kabbaoui et al. [92] explained that a high dose (500 mg/kg) of *Cistus ladaniferus* decreased the creatinine level in streptozotocin-induced diabetic rats. The leaf extract of *Anogeissus acuminata* also decreased the creatinine level in streptozotocin-induced diabetic rats as presented by Navale et al. [93]. Under diabetic conditions, due to an increase in glucose level, kidney damage occurs. This kidney damage leads to an increase in the urea level [94]. In the present study, a high dose of FXBH decreased the concentration of urea more in comparison to a low dose (Table 5). The results of our study have been supported by Nasry et al. [95] who explained that a high dose of petroleum ether extract (200 mg/kg) of *Gaur gum* decreases the serum level of urea in streptozotocin-induced diabetic rats.

Blood analysis evaluated the amount of glucose associated with hemoglobin, and the findings are measured using the hemoglobin A1c (HbA1c). The blood level of HbA1c was highest in diabetic rats compared to the control group (Table 5). HbA1c levels in diabetic rats administered a high dose of FXBH were noted to decline in comparison to the control group. Our results are in accordance with Kooti et al. [96] and Gurjar et al. [97], explaining that plant bioactive compounds have a pronounced role in reducing HbA1c level in diabetic rats.

This study also evidenced that the *n*-hexane fraction of *F. xanthoxyloides* bark possesses the ability to reduce the chances of developing diabetic complications and lower the risk of developing diabetes complications. Islets of Langerhans regeneration to normal is possible in diabetic rats treated with both low and high dosages (Fig 8). The FXBH, which prevents intestinal glucose absorption, is responsible for this impact. Following treatment with FXBH, the size of the islets of Langerhans reverted to normal, and the pancreatic cells were able to repair and regenerate. The histopathological observations presented here are qualitative in nature and based on microscopic examination. No morphometric measurements or histological scoring analysis were undertaken in this study. Our results are consistent with those of Mohan et al. [98] who used a methanolic extract of *Catharanthus roseus* to show a minor regeneration of β-cells in the alloxan-induced diabetic rats compared to the diabetic control group. Amraie et al. [99] also reported a similar result that explains that the control group demonstrated only a minor resistance to alloxan-induced alterations in pancreatic cells. For an increased number and size of islets of Langerhans, an alfalfa administration enhanced the quantity of insulin production. This work added to the evidence that the n-hexane fraction of *F. xanthoxyloides* bark possesses diabetes-lowering characteristics in it. More research should be done on the molecular mechanism of the strong bioactive compounds found in the *F. xanthoxyloides* plant. Numerous pharmacological aspects of *F. xanthoxyloides*, including appropriate dosage and clinical efficacy, remain unclear. More scientific research and safety profiling are needed in relation to current studies on *F. xanthoxyloides* bark extract in order to clarify and make the treatment and prevention of diabetes more evident. Interestingly, arrays of studies have proved the antidiabetic properties of other *Fraxinus* species. For example, the leaf extract of *F. angustifolia* (25 and 50 mg/kg b.w.) exhibited antidiabetic and hepatoprotective activity in streptozotocin (STZ)-induced diabetic rats and paracetamol-fed mice [100,101]. Another species, namely, *F. floribunda,* displayed significant α-glucosidase inhibition and antidiabetic activity in the STZ-induced diabetic model [102]. The administration of hydroethanolic extract from *F. ornus* (10 and 50 mg/kg b.w.) demonstrated significant antihyperglycemic properties in the nicotinamide-STZ-induced diabetic model [103]. Another study on the total flavonoids isolated from *F. mandshurica* highlighted the in *vitro* antidiabetic potential via α-glucosidase inhibitory effects supported with molecular docking experiments [104]. A double blind, randomized, controlled clinical trial registered the positive effects of *F. excelsior* seed extract on postprandial hyperglycemia and insulin release in the healthy volunteers [105].

## 5. Conclusion

Our research findings demonstrated that the *n*-hexane fraction of *Fraxinus xanthoxyloides* bark (FXBH) exhibited noteworthy antidiabetic properties by effectively inhibiting the α-amylase, α-glucosidase, and DPP4-inhibiting activity and high glucose uptake *in vitro* and reducing blood glucose levels and ameliorating various biochemical markers, including cholesterol, triglycerides, LDL, lipase, amylase, ALT, AST, creatinine, urea, and CRP *in vivo*. Additionally, FXBH exhibited significant antidiabetic potential by elevating serum high-density lipoprotein (HDL) levels while positively impacting cholesterol levels. Histopathological analysis of the pancreas demonstrated the restoration of β-cells in rats treated with FXBH. This analysis revealed that FXBH possesses potent antidiabetic potential, and to better understand the molecular mechanisms underlying *F. xanthoxyloides’* antidiabetic action, further pharmacokinetic and pharmacodynamic characteristics are required. Further studies are recommended to investigate the synergistic effects of the *F. xanthoxyloides* extract and standards antidiabetic drugs are recommended for availability to be used as adjuvant therapy

## Supporting information

S1 Fig Graphical abstract. (JPG)
