# Supplementary figures and images for: Protective effects of Fraxinus xanthoxyloides bark in alloxan-induced diabetic rats: A phytochemical and pharmacological approach
Source: PLoS One. 2026 Jun 4;21(6):e0346328. doi: 10.1371/journal.pone.0346328 (PMC13235879; doi:10.1371/journal.pone.0346328)

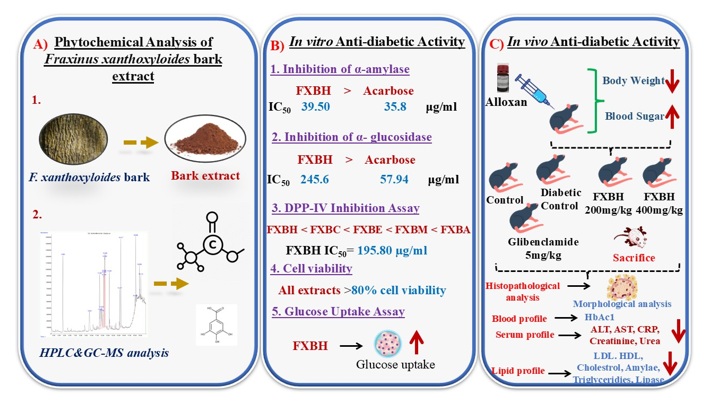

Supplement: S1 Fig — (JPG) [file pone.0346328.s001.jpg]
